# Maintenance therapy with capecitabine in patients with locally advanced unresectable pancreatic adenocarcinoma

**DOI:** 10.3892/ol.2014.2238

**Published:** 2014-06-11

**Authors:** MUHAMMAD WASIF SAIF, LESLIE LEDBETTER, KRISTIN KALEY, MARIE CARMEL GARCON, TERESA RODRIGUEZ, KOSTAS N. SYRIGOS

**Affiliations:** 1 Section of GI Cancers and Experimental Therapeutics, Tufts University School of Medicine, Boston, MA 02111, USA; 2 University of Alabama at Birmingham, Birmingham, AL 35233, USA; 3 Yale School of Medicine, New Haven, CT 06520, USA; 4 Columbia University Medical Center, New York, NY 10027, USA; 5 Oncology Unit, Third Department of Medicine, University of Athens, Sotiria General Hospital, Athens 115 27, Greece

**Keywords:** pancreatic cancer, capecitabine (Xeloda), radiotherapy, thymidine phosphorylase

## Abstract

Therapeutic options for locally advanced pancreatic cancer (LAPC) include concurrent chemoradiation, induction chemotherapy followed by chemoradiation or systemic therapy alone. The original Gastro-Intestinal Study Group and Eastern Cooperative Oncology Group studies defined fluorouracil (5-FU) with concurrent radiation therapy followed by maintenance 5-FU until progression, as the standard therapy for this subset of patients. Although this combined therapy has been demonstrated to increase local control and median survival from 8 to 12 months, almost all patients succumb to the disease secondary to either local or distant recurrence. Our earlier studies provided a strong rationale for the use of capecitabine in combination with concurrent radiation followed by maintenance capecitabine therapy. To report our clinical experience, we retrospectively evaluated our patients who were treated with maintenance capecitabine. We reviewed the medical records of patients with LAPC who received treatment with capecitabine and radiation, followed by a 4-week rest, then capecitabine alone 1,000 mg twice daily (ECOG performance status 2 or age >70 years) or 1,500 mg twice daily for 14 days every 3 weeks until progressive disease. We treated 43 patients between September 2004 and September 2012. The population consisted of 16 females and 25 males, with a median age of 64 years (range, 38–80 years). Patients received maintenance capecitabine for median duration of 9 months (range, 3–18 months). The median overall survival (OS) for these patients was 17 months, with two patients still living and receiving therapy. The 6-month survival rate was 91% (39/43), 1-year survival rate was 72% (31/43) and 2-year OS rate was 26% (11/43). Grade 3 or 4 toxicity was observed rarely: Hand-foot syndrome (HFS) in two patients, diarrhea in one patient and peripheral neuropathy in one patient, and there was no mortality directly related to treatment. Capecitabine maintenance therapy following chemoradiation in LAPC offers an effective, tolerable and convenient alternative to 5-FU. To the best of our knowledge, this is the largest study of its kind which has determined the safety and efficacy of capecitabine maintenance therapy for patients with LAPC.

## Introduction

In 2013, there will be an estimated 45,220 new cases and 38,460 mortalities attributed to pancreatic cancer in the United States (1). The prognosis of pancreatic cancer, regardless of stage, is extremely poor, with a 1-year survival rate of 25% and a 5-year survival rate of <5%. Only a small percentage of patients are able to undergo complete surgical resection with potential curative intent. Approximately 30–40% of patients with pancreatic cancer present with locally advanced, unresectable (LAPC) disease (2). Generally, pancreatic cancer is classified as unresectable in several cases; if there is evidence of involvement of the superior mesenteric artery or celiac axis, extrapancreatic involvement and/or metastatic disease (3). Tumor encasement or occlusion of the superior mesenteric vein or the superior mesenteric vein-portal confluence does not rule out resection, as some centers are demonstrating the feasibility of superior mesenteric vein reconstruction (4).

The treatment of LAPC requires a multidisciplinary approach. Surgery is not an optimum route in LAPC, as lesions have a high probability of incomplete surgical resection due to residual cancer at the surgical margin (5). Given the limitations of surgery for LAPC, chemotherapy combined with radiation has been utilized in an effort to improve local and distant tumor control (2). However, with these treatment options, the median survival of LAPC is ~8–12 months (2,6).

There are three prospective randomized trials comparing radiation to combined chemoradiation, all of which concluded differently from each other (Table I). In the pivotal Gastro-Intestinal Study Group (GITSG) study, 194 patients with LAPC were randomly assigned to high-dose (6000 rads) radiation therapy alone, to moderate-dose (4000 rads) radiation + 5-fluorouracil (5-FU) and to high-dose radiation plus 5-FU. The 5-FU was administered as a bolus at the dose of 500 mg/m^2^ bolus days 1–3 with concurrent radiation followed by maintenance 5-FU bolus 500 mg/m^2^/week until progression (7). Both 5-FU-containing arms produced a highly significant survival improvement when compared with radiation alone. Forty percent of patients treated with the combined regimens were still living at one year compared with 10% of patients treated with radiation only. Survival differences between 4000 rads plus 5-FU and 6000 rads plus 5-FU were not significant with an overall median survival time of 10 months. In the Eastern Cooperative Oncology Group (ECOG) trial, 191 patients with pathologically confirmed, locally unresectable adenocarcinoma of the stomach (57 patients) and pancreas (91 patients), were randomly allocated to therapy with 5-FU alone, 600 mg/m^2^ intravenously (IV) once weekly, or radiation therapy (4,000 rad) plus adjuvant 5-FU, 600 mg/m^2^ IV on the first three days of radiotherapy, then follow-up maintenance 5-FU, 600 mg/m^2^, weekly (8). The median survival time was similar for the two treatment programs and for both types of primary carcinoma, and was as follows: gastric primary carcinoma, 5-FU, 9.3 months; 5-FU plus radiotherapy, 8.2 months; pancreatic primary carcinoma, 5-FU, 8.2 months; 5-FU plus radiotherapy, 8.3 months. Substantially more toxicity was experienced by patients treated with the combined modality arm than by those patients receiving 5-FU alone. In the ECOG 8282 study, 104 patients were randomized to receive radiation (59.4 Gy) alone or radiation with concurrent infusional 5-FU (1,000 mg/m^2^/day as a continuous infusion on days 2–5 and 28–31) plus mitomycin (one-time bolus of 10 mg/m^2^ on day 2) (9). There was no benefit with the addition of chemotherapy as response rate (9 versus 6%), median disease-free survival (DFS; 5.0 versus 5.1 months) and overall survival (OS; 7.1 versus 8.4 months) times were similar, respectively. The lack of survival benefit observed in the ECOG study was attributed to the dose and method of 5-FU administration as well as the addition of toxicity secondary to mitomycin. Generally, it is believed that radiation alone is a suboptimal treatment for LAPC, as the majority of patients will succumb to systemic disease.

Since the conduct of the above trials, the combination of 5-FU and radiation therapy followed by maintenance 5-FU has been considered a standard care for locally advanced pancreatic cancer based on the Mayo Clinic and GITSG trial results. The majority of the older trials employed bolus 5-FU with radiation, but the later data suggested that infusional 5-FU offers a pharmacologically better and less toxic (myelosuppression) approach in combination with radiation. A phase I ECOG study demonstrated that concurrent radiation with protracted 5-FU infusion at 250 mg/m^2^/day was well tolerated with a median OS time of 11.9 months (11). Following this, a phase II study showed similar results when 20 patients with LAPC received protracted 5-FU infusion (200 mg/m^2^/day) with concurrent radiation (50.4 Gy in 28 fractions over 5.5 weeks) (12). The median OS time was 10.3 months. There is a lack of phase III data lacks in comparing bolus versus infusional 5-FU with radiation in LAPC.

Gemcitabine is the standard chemotherapy used in metastatic pancreatic cancer, for its demonstrated improvements in clinical benefit and survival compared with 5-FU (13). Gemcitabine also has potent radiation-sensitizing effects that has led investigators to evaluate the combination of gemcitabine and radiation in LAPC. There are multiple phase I and II trials using variable doses of gemcitabine, with different schedules and doses of radiation (Table II).

Capecitabine is a rationally designed oral fluoropyrimidine carbamate that is absorbed intact through the intestinal wall, and then converted to 5-FU in three sequential enzymatic reactions (18). During the third step, 5′-deoxy-5-fluorouridine is converted to 5-FU by the enzyme thymidine phosphorylase (TP) at the tissue level. TP is present in significantly higher concentrations in cancer cells than in plasma or surrounding normal tissue, so has an improved antitumor effect by producing a higher intratumoral concentration of 5-FU, while simultaneously sparing many of 5-FU’s associated systemic toxicities (19). Radiation has been shown to upregulate TP and hence lead to the production of more 5-FU within the tumor tissue (19). Pancreatic xenograft studies from our laboratory demonstrated a synergistic antitumor effect with concomitant capecitabine and radiotherapy for both radiated and contralateral lead-shielded tumors in the same animals (abscopal effects) (20). We pioneered the phase I study, which concluded that capecitabine 800 mg/m^2^ twice daily with concurrent radiation therapy is feasible in patients with LAPC (21). Compared with intravenous 5-FU, capecitabine is associated with a lower incidence and severity of a number of symptoms, including diarrhea, stomatitis, nausea and neutropenia, but it has been demonstrated to increase the rate of hand-foot syndrome (HFS). This approach offers a simple alternative to intravenous fluorouracil as a radiosensitizer. This was further confirmed in a phase II study by our research group (22) and, as a result, all the major cooperative research groups adopted capecitabine as a radiosenstitizer of choice in this setting.

While combined modality in LAPC is generally well accepted, data on maintenance therapy is poorly established in this setting. The concept of maintenance therapy in LAPC dates back to the pivotal GITSG and ECOG studies, which used weekly bolus 5-FU until progressive disease. Substantial evidence suggests that chronically administered capecitabine is feasible in breast cancer patients (23), and has demonstrated that intravenous 5-FU is safe and non-GI tumors (OPTOMIX2 study) have antitumor activity in gastro-intestinal (GI) (24). However, the choice of infusional 5-FU as a maintenance agent is cumbersome, whereas oral 5-FU derivatives may offer a more feasible and convenient alternative.

We report a retrospective analysis of the efficacy and toxicity of capecitabine as a long-term maintenance therapy in patients with LAPC treated during September 2004 through to September 2012.

## Materials and methods

We reviewed records of 43 patients with LAPC who were treated at the Tufts University School of Medicine (Boston, MA, USA), University of Alabama at Birmingham (Birmingham, AL, USA), Yale School of Medicine (New Haven, CT, USA) and Columbia University Medical Center (New York, NY, USA) with capecitabine monotherapy after completing capecitabine with concurrent radiation. Information regarding patient characteristics, treatment duration and dosage, toxicity and survival was obtained from medical charts and through the tumor registry. Patients received capecitabine alone 1,000 mg twice daily [ECOG performance status (PS) 2 or age >70 years] or 1,500 mg twice daily for 14 days every 3 weeks until progressive disease. Survival was measured from date of treatment initiation. Disease response was measured according to the RECIST criteria (25). Assessments of tumor dimensions were performed prior to treatment and then every three cycles (9 weeks). Patients were assessed every 3 weeks during capecitabine monotherapy. Toxicity was assessed per NCI-CTCAE v3.0 (26).

Patient characteristics were analyzed with frequency tables, with groupings assigned to a respective percentage of the entire data set. Toxicity was analyzed using frequency tables with groupings assigned to a respective percentage of the entire data set. Survival was assessed with Kaplan-Meier survival analysis and was measured from date of diagnosis until date of mortality or, for surviving patients, through to the end of the study period.

## Results

### Patient characteristics

The population consisted of 16 females and 25 males, with a median age of 64 years (range, 38–80 years). Five patients were aged >70 years. Fourteen patients were of ECOG PS 2, 22 patients were of ECOG PS 1 and seven patients were of ECOG PS 0. The most common symptom leading to upgrading the ECOG status was pain associated with disease. Forty one patients received initial treatment with a radiosensitizing dose of capecitabine of 800 mg/m^2^ from Monday through Friday with concurrent radiation; one with infusional 5-FU (reimbursement issue related to capecitabine) and one with gemcitabine for the first three weeks and later changed to capecitabine due to neutropenia. All the patients had recovered from any toxicity prior to starting the maintenance capecitabine monotherapy. Median time to start monotherapy capecitabine was 4 weeks (range, 4–7).

### Toxicity

Grade 3 or 4 toxicity was observed rarely: HFS in two patients, diarrhea in one patient, and peripheral neuropathy in one patient (Table III). There was no death directly related to treatment. During capecitabine treatment alone, grade 1 to 2 anemia was also observed, but was not different from that demonstrated in other trials. Leukocytes showed no marked decrease, with the median values remaining marginally below or above the lower limit of the normal range (4.0/nl) throughout the entire study period. Platelet counts demonstrated a similar decrease, again with no trend toward cumulative toxicity. Counts below the normal range were rare.

### Treatment delays and modifications

Capecitabine treatment was withheld from the patients who developed grade 3 toxicities as well as in those who developed grade 2 HFS or grade 2 GI toxicities, including diarrhea. Supportive management was provided. Capecitabine cycles were held from 1 to 4 weeks with resolution of symptoms. Six patients were administered reduced doses as a consequence of adverse symptoms: Two patients for grade 3 HFS, one patient for grade 3 diarrhea and three patients for fatigue and anorexia, while one patient was completely withdrawn from the capecitabine treatment due to peripheral neuropathy.

Capecitabine was continued as treatment until disease progression or unacceptable toxicities as mentioned earlier. The most common areas of progression included liver, peritoneum, distant lymph nodes and lungs. The most common agents used after failing capecitabine included gemcitabine-oxaliplatin (60%), gemcitabine-cisplatin (10%) and gemcitabine as a single agent (30%).

### Overall response

With continued capecitabine therapy, two patients with LAPC were thought to be radiologically converted to a resectable disease. Both were taken to surgery; laparoscopic examination showed peritoneal metastasis in one patient and the second patient had resection of the tumor with positive margins. In addition to these two patients, an additional four patients had a partial response (PR), totaling a response rate of 14%. Sixteen patients had stable disease for a median duration of nine cycles (27 weeks) during treatment with capecitabine alone. Five patients progressed at the time of first imaging in 9 weeks.

### Survival

Patients received maintenance capecitabine for a median duration of 9 months (range, 3 to 18 months). Survival was measured from date of initial treatment until date of mortality or until the study period concluded. The median OS time for these patients was 17 months (range, 3 to 21 months), with two patients still alive and on capecitabine maintenance therapy. The 6-month survival rate was 91% (39/43), 1-year survival rate was 72% (31/43) and 2-year OS rate was 26% (11/43).

## Discussion

The major benefit of capecitabine lies in its favorable toxicity profile. Multiple studies of capecitabine in the treatment of various GI malignancies have demonstrated that the incidence of GI toxicity and HFS is comparable to that of infusional 5-FU (27–29). This study reviewed a number of different tumor types and the treatment period with capecitabine was during radiation only, without analyzing ongoing treatment with cycles of capecitabine monotherapy. Our study also indicated a favorable toxicity profile with maintenance capecitabine therapy. Toxicity comparisons of 5-FU and capecitabine are perhaps best analyzed in studies conducted in colorectal and gastroesophageal malignancies (27,30). A phase III study comparing oral capecitabine to 5-FU in colorectal cancer reported significantly fewer occurrences of diarrhea, stomatitis, nausea, alopecia and grade 3/4 neutropenia with capecitabine. Although grade 3 HFS and grade 3/4 hyperbilirubinemia were more frequent with capecitabine (31).

For the sake of simplicity, tolerability and convenience, we selected a continuous fixed dose of capecitabine as a maintenance therapy for treatment of patients with LAPC. In clinical practice, the FDA-approved dose (http://gemcit-abine.com/fda_info.htlm) for metastatic colorectal and breast cancer is 2,500 mg/m^2^ divided into two equal daily doses for the first 2 weeks of a 3-week cycle. This dose is rarely used due to intolerable, dose-limiting toxicities, particularly HFS and diarrhea. Our data not only followed the GITSG study in LAPC patients but also complements studies conducted with novel oral 5-FU derivatives in other malignancies. One such example is the use of UFT (uracil and tegafur) tested in patients with lung cancer and gastric cancer (32,33).

We reviewed the literature and identified similar data in 10 patients with pancreatic cancer who were safely treated with maintenance capecitabine by Sun *et al* (34). However, in this case, a different schedule of capecitabine was used: 1,000 mg twice daily, Monday through Friday, with Saturday and Sunday off. Our regimen is more consistent with the FDA’s approved schedule. In addition, in this study we included only patients with LAPC. The toxicity profile was very favorable and consistent, with the exception of one patient who developed foot drop. Work-up was done and we believed it to be associated with capecitabine-induced peripheral neuropathy, similar to our previous experience (35).

It is important to acknowledge that capecitabine is possibly the most common agent also used in the treatment of metastatic pancreatic cancer. One study evaluated this in patients with metastatic or unresectable, locally advanced pancreatic cancer (36). Among 42 patients, 7.3% had PR. The major pitfall, as associated with any oral agent, is in patients controlling their medication. By using a simple and convenient dosing schedule, such mistakes can be reduced.

In conclusion, capecitabine is advantageous, in that it can be safely and conveniently administered as a maintenance therapy in patients with LAPC. Our study has provided the largest number of patients in this setting. Oral capecitabine has good bioavailability, did not have problems associated with intolerance of oral medication and had a tolerable side-effect profile. Tumor response and survival were comparable if not better than with standard treatment with intravenous 5-FU.

## Figures and Tables

**Table I:** Radiation therapy versus chemoradiation.

Series	Number of patients	Median survival time (months)	1-year survival (%)	Ref.
Mayo Clinic				(10)
XRT (35–40 Gy/3–4 weeks) only	32	6.3	6	
XRT (35–40 Gy/3–4 weeks) + 5-FU	32	10.4	22	
GITSG				(7)
XRT (60 Gy/10 weeks) only	25	5.3	10	
EBRT (40 Gy/6 weeks) + 5-FU	83	8.4	35	
EBRT (60 Gy/10 weeks) + 5-FU	86	11.4	46	
ECOG				(9)
EBRT (59.4 Gy/five 1.8-Gy fractions/week)	49	8.2	NR	
EBRT + 5-FU and mitomycin (10 mg/m^2^ on day 2)	55	8.2	NR	

NR, not reported; XRT, radiation therapy; EBRT, external beam radiation therapy; GITSG, Gastro-interstinal Study Group; ECOG, Eastern Cooperative Oncology Group.

**Table II:** Phase II–III trials using gemcitabine with radiation.

First author (ref.)	Regimen	Number of patients	Median survival time (months)	1-year survival (%)
Blackstock *et al* (14)	Twice-weekly gemcitabine at a 40 mg/m^2^ dose + concurrent XRT (50.4 Gy)	39	8.2	NR
Moore *et al* (15)	Weekly gemcitabine (600 mg/m^2^) + concurrent XRT (50.4 Gy)	28	7.9	31.1
Epelbaum *et al* (16)	Gemcitabine 400 mg/m^2^ weekly x3 every 28 days + concurrent XRT (50.4 Gy)	20	8	NR
Haddock *et al* (17)	Gemcitabine 30 mg/m^2^ twice weekly and cisplatin 10 mg/m^2^ + concurrent XRT (50.4 Gy)	20	8.8	29

NR, not reported; XRT, radiation therapy.

**Table III:** Toxicities during capecitabine maintenance.

	Grade of toxicity			
	
Adverse event	1	2	3	4
HFS	9	4	2	0
Diarrhea	11	6	1	0
Vomiting	5	1	0	0
Nausea	7	2	0	0
Mucositis	3	1	0	0
Hyperbilirubenmia	5	2	0	0
Anorexia	6	3	0	0
Fatigue	5	4	0	0
Neutropenia	2	1	0	0
Anemia	3	2	0	0
Thrombocytopenia	1	0	0	0
Peripheral neuropathy	3	0	1	0

HFS, hand-foot syndrome.
